# Therapy-educated fibroblast states link treatment pressure to residual disease and acquired resistance in gastric cancer

**DOI:** 10.3389/fimmu.2026.1958611

**Published:** 2026-09-15

**Authors:** Zhigang Chen, Yongtao Yang, Ting Yang, Liping Gao, Ting Zhou, Mengyu Deng

**Affiliations:** 1 Department of Gastroenterology, The 940th Hospital of Joint Service Logistics Support Force of Chinese People's Liberation Army, Lanzhou, China; 2 Department of Anesthesiology and Operating Room, The Second Hospital of Lanzhou University, Lanzhou, China; 3 Departments of Nursing and Neurology, and General Medicine Ward, Honghe Hospital Affiliated to Kunming Medical University (South Yunnan Central Hospital of Yunnan Province), Gejiu, Yunnan, China; 4 Department of Science and Education, Jiangbin Hospital of Guangxi Zhuang Autonomous Region (The Third People’s Hospital of Guangxi Zhuang Autonomous Region), Nanning, China

**Keywords:** acquired resistance, cancer-associated fibroblasts, gastric cancer, immune evasion, immunotherapy, residual disease, therapy-induced plasticity, tumor microenvironment

## Abstract

Chemotherapy, immune checkpoint blockade, HER2-targeted therapy, and anti-angiogenic therapy have widened treatment options for gastric cancer, yet residual disease and acquired resistance remain common. Cancer-associated fibroblasts (CAFs) are often classified as fixed subtypes, even though treatment exposes them to tissue injury, inflammatory cytokines, hypoxia, altered interstitial pressure, and matrix stress. We use treatment-shaped fibroblast state to describe a functional program that arises through reprogramming within resident fibroblasts, recruitment from a local mesenchymal source, or enrichment of a pre-existing state that survives therapy. An unpaired change in abundance cannot distinguish these mechanisms. The review follows four time points: the baseline ecosystem, acute therapeutic stress, the residual disease niche, and the acquired resistance circuit. Interferon-responsive and senescence-associated programs may be short-lived and either recruit or suppress immune cells. By contrast, persistent matrix, vascular, metabolic, and paracrine programs can protect residual tumor cells during treatment. Gastric cancer studies implicate TGF-β-SMAD, IL-6-JAK-STAT3, GAS6-AXL, CXCL12-CXCR4, NRP2, LOX, FAK, and extracellular matrix pathways. Longitudinal human evidence is limited and is sensitive to biopsy timing, spatial sampling, and single-cell dissociation bias. Translational studies should pair spatial specimens with collagen-turnover and extracellular-vesicle biomarkers, mechanically matched organoid-CAF co-cultures, and immunocompetent or humanized models. Treatment should disrupt or reprogram the state that sustains residual disease without removing fibroblast functions that support immunity or restrain tissue injury.

## 1 Introduction

Current gastric cancer (GC) treatment combines fluoropyrimidine-platinum chemotherapy with immune checkpoint inhibitors (ICIs), HER2-targeted agents, or anti-angiogenic therapy according to setting and biomarkers, with ramucirumab remaining a later-line option (1). PD-1 blockade plus chemotherapy improves first-line outcomes in HER2-negative disease, while trastuzumab established HER2-directed therapy (2, 3). Nevertheless, radiographic response can leave viable cells within remodeled stromal, vascular, and immune niches.

Resistance studies usually focus on tumor genetics, antigenicity, and bypass signaling. Therapy, however, also changes the tissue on which surviving cells depend. CAF programs affect drug penetration, immune entry, paracrine survival, and matrix repair. Consensus guidance defines a CAF state by its molecular features, location, and function rather than by one marker (4). In this review, transition denotes a program change within the same lineage. Reprogramming is treatment-dependent and potentially reversible, selection is survival or expansion of a pre-existing program, and recruitment is the entry or local conversion of another mesenchymal source. Changes in cell abundance in unpaired scRNA-seq data establish remodeling, but they do not identify which of these processes occurred.

Gastric cancer atlases resolve myofibroblastic or matrix-producing, inflammatory, perivascular, proliferative, and metabolically supportive programs. Matrix-producing CAFs often express ACTA2, TAGLN, COL1A1/COL3A1, FAP, or INHBA, whereas inflammatory CAFs show IL6, CXCL12, CCL2, and related cytokine programs (5, 6).

Antigen-presenting CAFs express MHC-II antigen-presentation machinery, including HLA-DRA and CD74, and may support local T-cell activation or tolerance depending on co-stimulation and spatial context (7).

Other gastric cancer studies describe IGF1-rich and LOX-positive programs (8, 9). CCDC80-positive and PDPN-positive programs have also been reported (10, 11).

GPC3-high and THBS4-rich programs have likewise been described (12, 13). These labels overlap and remain cohort-dependent. Across gastric cancer studies, CAFs communicate through cytokines, chemokines, extracellular-matrix proteins, and extracellular vesicles rather than through a single defining secretome (4, 6). The following sections consider how treatment changes the abundance, function, and persistence of these programs.

## 2 Baseline fibroblast states that condition treatment response

Pretreatment GC contains fibroblast programs that can condition therapy. Single-cell and spatial atlases identify FAP/INHBA-enriched, matrix-remodeling, inflammatory, contractile, proliferative, and antigen-presentation programs, with variation across histologic subtypes. Marker combinations are not interchangeable between cohorts (5, 6).

Baseline states can define functional niches. IGF1-rich fibroblasts have been associated with inferior chemotherapy response and recurrence, while LOX-positive fibroblasts associate with suppressive macrophages and IL-6 signaling (8, 9). CCDC80-positive regions associate with CD8 T-cell dysfunction (10). These data do not establish immutable lineages.

TGF-β and matrix stiffness favor contractile fibroblast programs, whereas inflammatory cytokines and chemokines connect CAFs with myeloid cells and lymphocyte positioning (4). VEGF, hypoxia, collagen, and hyaluronan jointly alter perfusion, solid stress, interstitial fluid pressure, and drug transport. In gastric tissue, stiffness increases from approximately 0.5–1 kPa in non-malignant tissue to as much as 7 kPa in tumors, with associated integrin-FAK-SRC and YAP/TAZ activation (14, 15). Tumor killing may transiently relieve stress, while edema, fibrosis, vessel pruning, and repair may restore or worsen it. Spatial ligand mapping, perfusion, elastography, and perturbation therefore provide more information than bulk stromal scores.

CAF composition varies with biopsy depth, tumor region, Lauren histology, molecular subtype, and stage. Superficial biopsies may miss deep desmoplasia or perivascular states, while ulceration may introduce wound-repair fibroblasts. Pretreatment signatures are spatial samples: they may identify a pre-existing niche but cannot show what therapy will induce (5, 6).

Baseline CAF labels are most useful when they point to a testable mechanism, such as a collagen barrier, a CXCL12-rich exclusion zone, or a poorly perfused perivascular niche. They should not be read as fixed lineages.

## 3 Treatment-shaped fibroblast states in gastric cancer

### 3.1 Cytotoxic chemotherapy

Cytotoxic therapy produces fibroblast injury and wound-repair signals. A small longitudinal gastric cancer study found increased fibroblast and endothelial fractions after neoadjuvant chemotherapy, but only three patients had paired samples, supporting remodeling rather than lineage transition (16).

Gastric cancer models link residual disease to TGF-β-SMAD3/ECM programs and NRP2-dependent CAF protection (17, 18).

Other models implicate GAS6-AXL-STAT3-ABCG1 drug efflux and TGF-β-driven HAPLN1/collagen remodeling (19, 20). Together, these findings support repair- and matrix-biased states, not one conserved patient trajectory.

Biopsy timing is a biological variable, not merely a reporting detail. As a pragmatic operational framework, we propose four sampling bins: baseline; acute stress, ideally 24 hours to 14 days after treatment begins and before substantial clonal outgrowth; residual disease after several cycles or at resection; and acquired resistance at protocol-defined progression. These cut-offs are intended for study design and have not been clinically validated. Acute samples may capture DNA damage, transient NF-κB signaling, interferon exposure, edema, or a senescence-associated secretory phenotype. Samples obtained weeks or months later contain cells that have survived repeated treatment and completed matrix repair. A persistent treatment-educated state should be present at two or more on-treatment time points, remain spatially associated with viable residual tumor, and show functional dependence in withdrawal or perturbation experiments. Reports should give the interval from the last dose, cumulative exposure, corticosteroid use, histologic regression, ulceration, and background fibrosis.

### 3.2 Immune-checkpoint blockade and immunochemotherapy

Immunochemotherapy combines interferon-associated inflammation with cytotoxic tissue injury. A prospective neoadjuvant tislelizumab-SOX study identified three post-treatment CAF phenotypes, with greater inflammatory CAF abundance in poor responders. The temporal comparison was not fully paired (21).

PDPN-positive and GPC3-high CAF programs were associated with weaker response to immunotherapy (11, 12), while gastric cancer models link CAF-derived LOX to suppressive programs (22). These findings do not make every inflammatory or interferon-responsive CAF harmful.

Cross-cancer and gastric cancer studies indicate that MHC-II-positive fibroblasts can support local CD4 T-cell responses when antigen presentation occurs in an immune-permissive spatial context (7, 23). Early after immunochemotherapy, such a program may assist T-cell recruitment or activation. By contrast, other CAF programs are associated with poor ICI response and immune exclusion (11, 12). Responders and non-responders should therefore be compared at matched time points using spatial measures of CD8 entry, dendritic-cell activation, tertiary lymphoid organization, and Treg or myeloid proximity; average MHC-II or inflammatory-marker abundance alone is insufficient.

### 3.3 Targeted and anti-angiogenic therapies

Targeted therapies can reshape the stroma through target loss, payload distribution, and perfusion changes. CAF-derived THBS4 reduces ERBB2 expression and promotes trastuzumab resistance in gastric cancer models. Conventional trastuzumab remains dependent on regional HER2 availability and tissue access, both of which can be limited by CAF-driven receptor downregulation and dense ECM (13, 14).

Trastuzumab deruxtecan (T-DXd) differs because internalization and linker cleavage release a membrane-permeable topoisomerase-I payload that can diffuse into adjacent HER2-low or HER2-negative cells and produce local bystander killing in heterogeneous tumors. DESTINY-Gastric01 showed clinical activity after trastuzumab (24, 25). A CAF-rich matrix may still restrict antibody delivery and create hypoxic, slowly cycling compartments, but it need not abolish the short-range bystander effect once enough ADC reaches HER2-positive cells. Comparative studies should measure regional ADC penetration, HER2 mosaicism, ECM density, and payload pharmacodynamics instead of extrapolating trastuzumab resistance directly to T-DXd.

Gastric cancer studies implicate both angiogenic CAF programs and dynamic plasma VEGF-pathway markers during ramucirumab-paclitaxel (26, 27). Neither observation proves a longitudinal CAF transition.

Anti-angiogenic therapy can alter CAF function without increasing abundance. Cross-cancer models suggest that transient vascular normalization improves delivery and immune entry, whereas sustained pruning can increase hypoxia, matrix tension, and compensatory angiogenesis (14). GC studies identify angiogenic CAFs but have not shown this sequence longitudinally (26). Paired perfusion, vascular, ECM, and fibroblast measurements are needed before post-treatment hypoxia can be attributed to CAF-driven resistance.

### 3.4 Mechanical feedback during therapy

Therapy alters biophysical cues over time. Tumor death may decompress vessels, whereas edema raises interstitial pressure. Repeated deposition and LOX cross-linking increase stiffness and integrin-FAK/YAP-TAZ signaling, stabilizing contractile myCAFs. Prolonged vessel pruning may intensify hypoxia and matrix tension (14, 15). Because transport differs among small molecules, antibodies, ADCs, and immune cells, longitudinal studies should combine CAF states with elastography or rheology, collagen architecture, perfusion or pressure surrogates, and regional drug distribution.

The evidence in this section separates reversible acute stress from states that are selected or stabilized during treatment. A claim of transition still requires paired temporal, lineage, and functional evidence.

## 4 CAF-organized residual disease niches

Residual disease includes viable tumor cells and their persistent microenvironment. CAF extracellular vesicles can transmit HSF1-dependent programs, while IL-6 activates tumor JAK2-STAT3 (28, 29).

Lumican supports integrin β1-FAK signaling, while hyaluronan-CD44 promotes resistant peritoneal niches (30, 31).

CXCL12 promotes PD-L1 expression and inhibitory macrophage polarization, while spatial data associate SPP1-positive/C1QC-positive macrophages and exhausted CD8 T cells with ICI resistance (32, 33). Although mostly non-serial, these studies support a composite niche of dense matrix, abnormal vessels, CAF ligands, Tregs, suppressive myeloid cells, and dysfunctional cytotoxic cells.

Spatial structure matters because small residual nests may remain protected even when total stromal abundance falls. Collagen geometry can lengthen vessel-to-tumor transport routes, whereas lumican-integrin β1-FAK signaling provides adhesion-dependent survival (14, 30). CAF-derived extracellular vesicles can also transfer stress-response programs (28). Perivascular CAFs may influence local drug exposure, while marginal CAFs influence immune entry. Regional drug imaging, matrix mechanics, and spatial metabolomics are therefore more informative than total collagen staining.

### 4.1 Primary gastric CAFs versus peritoneal and omental fibroblasts

Peritoneal carcinomatosis creates a stromal compartment distinct from the primary gastric wall. Primary GC CAF programs arise within gastric-wall niches, whereas peritoneal and omental lesions develop in a mesothelial, adipose-rich, and ascitic environment. TGF-β-induced mesothelial-to-mesenchymal transition, marked by loss of E-cadherin and gain of α-SMA and vimentin, and tumor-associated mesothelial cells have been linked to fibrosis and peritoneal dissemination (34, 35). Hyaluronan-CD44 signaling defines an immunosuppressive, treatment-resistant niche in gastric cancer peritoneal metastasis (31). Accordingly, a CAF signature from the primary tumor should not be assumed to represent peritoneal implants; paired primary, ascites or lavage, omental, and implant sampling is needed to distinguish dissemination-associated recruitment from treatment-shaped states.

A residual niche is defined by viable tumor, fibroblast function, matrix continuity, vascular access, and immune penetration. Total CAF abundance alone does not justify depletion.

CAF-rich tissue is not uniformly immunosuppressive. Immunostimulatory states predict checkpoint response in another cancer, while antigen-presenting CAFs provide a gastric cancer-specific immune-supportive example (7, 36). These findings argue against indiscriminate depletion. Evidence still relies heavily on models and postoperative specimens, which may underrepresent treatment-sensitive regions. **Figure 1** summarizes progression from baseline ecology to a resistance circuit.

**Figure 1:**
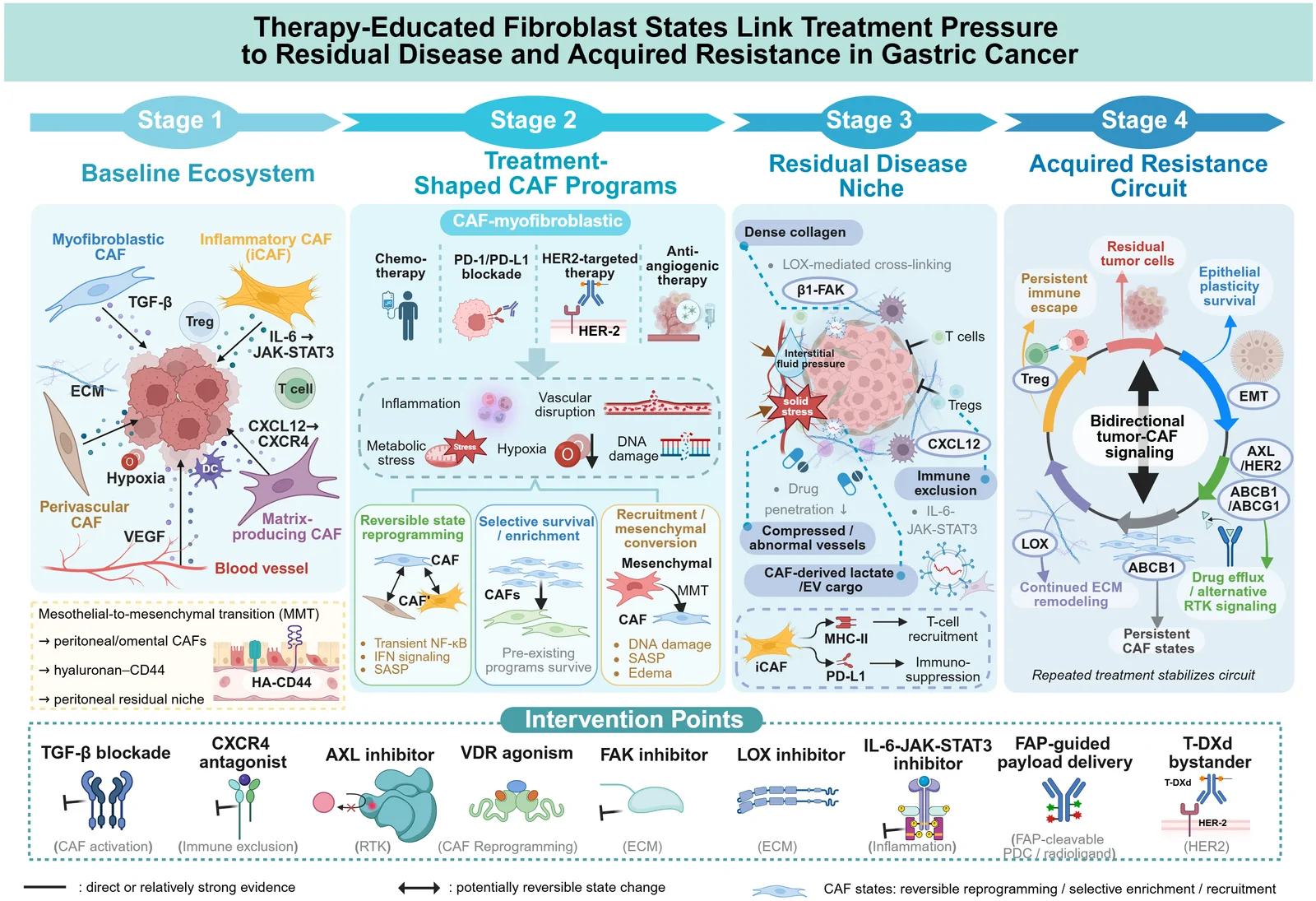
Sequential framework linking treatment pressure to residual disease and acquired resistance through therapy-educated fibroblast states.

## 5 From residual disease to acquired resistance

Residual cells are not synonymous with acquired resistance. Reversible chromatin states can precede stable resistance (37), while damaged tumor and stromal cells exchange survival factors.

Cross-cancer studies show that treatment-induced secretomes can support neighboring resistant cells and that DNA-damaged fibroblasts can release WNT16B (38, 39). These mechanisms are not proven in gastric cancer but explain how transient repair could become persistent stromal dependence.

A possible GC sequence begins with selection of stress-tolerant tumor cells that release TGF-β, IL-6-family cytokines, vesicles, or growth factors. CAFs then reinforce integrin-FAK signaling, alternative receptor-tyrosine kinases, plasticity, survival, and efflux (19, 30), while CXCL12/LOX-related matrix change, vascular dysfunction, and myeloid recruitment restrict cytotoxic cells (22, 32). Repeated treatment may stabilize this reversible protection into bidirectional tumor-CAF resistance, but this remains an integrative model rather than a lineage-resolved patient sequence.

For this review, we define primary and acquired resistance operationally. For advanced disease, primary resistance should denote progressive disease at the first protocol-defined assessment or failure to achieve a prespecified minimum clinical-benefit interval despite adequate exposure. Acquired resistance should require documented initial sensitivity, such as a complete or partial response or durable stable disease for a protocol-defined interval, followed by RECIST progression during treatment or shortly after discontinuation. For neoadjuvant therapy, residual viable tumor at resection should not be labeled acquired resistance without evidence of earlier regression and subsequent regrowth; postoperative recurrence should be analyzed separately. A fibroblast program can be called acquired only when it was absent or quantitatively minor at baseline, increases before or at progression in paired, anatomically matched tissue, and contributes functionally to resistance. If a program is already abundant before treatment and merely persists, the appropriate term is pre-existing or selected primary resistance. If baseline detection is limited by sampling, the conclusion should remain indeterminate. A pan-cancer single-cell atlas identifies recurrent fibroblast programs and inferred activation trajectories (40), but trajectories reconstructed from unpaired specimens cannot establish temporal conversion.

Treatment order may shape cross-resistance. A matrix selected during chemotherapy may impede immune-cell entry during later checkpoint blockade, while therapy-associated matrix tension and LOX signaling can reinforce hypoxia and immune exclusion (14, 22).

Treatment-shaped states may create targetable dependencies on AXL or FAK (19, 30). A credible dependency should increase during therapy, reduce residual-cell fitness when selectively inhibited, and weaken after treatment withdrawal. Organoid-CAF-immune cultures and serial *in vivo* models can test causality, but paired human pharmacodynamic data are still required to establish clinical relevance.

Acquired resistance is longitudinal, requiring adequate exposure, initial benefit, defined progression, and paired spatial or functional evidence that separates induction, selection, recruitment, and sampling variation.

## 6 Translational opportunities and unresolved questions

### 6.1 Phase-specific intervention, CAF-targeting strategies, and clinical activity

Translational studies should be phase-specific. Baseline matrix, immune penetration, and CAF programs may identify protected tumors; early biopsies, imaging, and blood markers can detect repair or exclusion; and residual lesions may provide a window for short, mechanism-matched intervention. This is a trial-design proposal, not established practice.

CAF-directed strategies can block TGF-β or CXCR4 signaling and, where mechanistically justified, inhibit AXL; none has been validated as CAF-directed therapy in gastric cancer (19, 41).

Cross-cancer studies link TGF-β blockade to reduced immune exclusion (42, 43) and CXCL12-CXCR4 or FAK inhibition to improved immune access (44, 45). These findings support mechanism-matched pathway or matrix intervention rather than marker-based CAF depletion.

The vitamin D receptor agonist calcipotriol provides a potential CAF-reprogramming strategy and attenuated CAF-mediated oxaliplatin resistance in preclinical gastric cancer co-culture models (46). FAP-cleavable faridoxorubicin releases doxorubicin in FAP-rich tissue (47).

FAP heterogeneity and injury to repair stroma remain practical safety concerns (41). More broadly, indiscriminate depletion is risky: removing α-SMA-positive fibroblasts or stromal Hedgehog signaling accelerated pancreatic cancer progression (48, 49). GC differs in anatomy, subtype, immunity, and treatment, so these findings are not direct GC evidence. Before depleting FAP-, ACTA2-, or PDPN-positive cells, trials should establish that the target is dispensable for vascular integrity, tissue restraint, antigen presentation, and repair. Short, state-selective reprogramming or ligand blockade may be safer.

Current activity remains early-phase or diagnostic. A gastric cancer FAP-PET study combines imaging with laparoscopy, whereas LuMIERE evaluates 177Lu-FAP-2286 in FAP-avid tumors (50, 51).

Faridoxorubicin tests FAP-released doxorubicin, and LY4337713 includes participants with FAP-positive gastric cancer (47, 52). These studies do not establish gastric cancer efficacy but illustrate target confirmation, delivery measurement, stromal pharmacodynamics, and tumor-specific efficacy and safety endpoints.

The useful therapeutic unit is a dependency present at a defined treatment phase, rather than a universal CAF marker. **Table 1** links the proposed states to their evidence, mechanisms, and candidate interventions.

**Table 1:** Therapy-educated fibroblast states in gastric cancer and their implications for residual disease and treatment resistance.

Treatment	Therapy-associated fibroblast state	Inducing signals	Immune and matrix effects	Role in residual disease or resistance	Evidence level	Potential intervention	Representative target drugs & clinical candidates
Cytotoxic therapy	Repair- and ECM-remodeling programs; fibroblast enrichment	DNA damage, TGF-β, IL-6, GAS6-AXL	Matrix deposition, drug-efflux support, reduced lymphocyte representation	Protects drug-tolerant cells and limits penetration	Paired human scRNA-seq (16); GC chemoresistance models (17, 19)	Early TGF-β/AXL blockade or matrix normalization	Galunisertib or bintrafusp alfa (TGF-β) (41); bemcentinib (AXL) (60)
ICI	PDPN-positive or GPC3-high programs; LOX-rich immune exclusion (emerging)	IFN-related inflammation, LOX, hypoxia	CD8/NK dysfunction, Treg and myeloid support	Maintains immune-excluded residual nests	Human response cohorts (11, 12); GC LOX model (22)	State-guided CXCR4, LOX, or communication blockade	Plerixafor (CXCR4) (41); preclinical LOX blockade (22)
Chemo-ICI	Inflammatory CAF enrichment associated with nonresponse	Combined tissue injury and checkpoint-driven inflammation	Mixed repair, antigen-presentation, and suppressive signaling	May couple cytotoxic persistence to immune escape	Prospective human trial-associated single-cell/spatial data (21)	Early paired monitoring and time-sequenced combinations	Calcipotriol (preclinical GC chemosensitization) (46)
HER2-targeted	THBS4-rich fibroblast signaling and HER2 downregulation	Tumor-CAF feedback; alternative survival signals	Stromal remodeling; apparent HER2 heterogeneity	Enables survival despite trastuzumab exposure	GC THBS4 co-culture/mouse models; no longitudinal human CAF validation (13)	Interrupt THBS4 signaling; reassess HER2 dynamically	T-DXd (bystander-capable HER2 ADC) (24, 25); HER2 reassessment
Anti-angiogenic therapy	Perivascular/angiogenic fibroblast programs (proposed)	VEGF-pathway perturbation, hypoxia, YAP/TAZ	Abnormal perfusion, myeloid recruitment, matrix stress	Reduces delivery and selects hypoxia-tolerant cells	Human biomarker dynamics; limited CAF-specific longitudinal evidence (26, 27)	Vascular/ECM normalization with toxicity-aware timing	Ramucirumab (therapy context) (27); matrix normalization remains investigational
FAP-high residual niche	FAP-enriched activated or matrix-remodeling programs	TGF-β, tissue injury, chronic fibrosis	Payload localization; possible loss of restraining or repair fibroblasts	May concentrate therapy in CAF-rich lesions; efficacy in GC unproven	GC imaging studies; pan-tumor early-phase treatment trials (47, 50)	FAP-guided imaging, cleavable drug conjugates, or radioligands	Faridoxorubicin/AVA6000 (47); 177Lu-FAP-2286 and LY4337713 (51, 52)
Peritoneal disease	MMT-derived, omental, and HA-rich fibroblast-like programs	TGF-β-SMAD2, ascitic cytokines, adipose-stromal cues	Adhesion, fibrosis, immune suppression, HA-CD44 signaling	Supports implantation and treatment-resistant peritoneal niches	MMT and peritoneal studies (31, 34); tumor-associated mesothelial model (35)	MMT/HA-CD44 blockade; compartment-specific delivery	HA-directed approaches remain investigational (41)

Evidence levels distinguish paired or treatment-associated human observations, human cross-sectional data, gastric cancer models, and cross-cancer inference. Proposed states should not be interpreted as proven lineage transitions without longitudinal or lineage-tracing evidence.

### 6.2 Validation models, liquid biopsy, and feasible sampling

Single-cell studies need explicit technical safeguards. Fibroblasts can be under-recovered because enzymatic disaggregation releases cell types unevenly and may not preserve fibroblast viability. Dense, cross-linked myofibroblastic regions may be especially prone to incomplete release, prolonged digestion, and viability filtering, although this remains an inference unless recovery is compared with intact tissue (53). Apparent enrichment of PDPN-positive, GPC3-positive, or CCDC80-positive states may reflect differential recovery as well as biology. Studies should report tissue mass and depth, ischemic time, enzyme cocktail, digestion duration, viability thresholds, and cells recovered per milligram. Matched histology, multiplex imaging, spatial transcriptomics, or single-nucleus RNA sequencing can estimate missing ACTA2-positive, collagen-rich, or perivascular compartments.

Causal validation should use autologous 3D patient-derived gastric cancer organoid-CAF-immune co-cultures rather than fibroblasts maintained only on supraphysiologically stiff two-dimensional plastic. Tunable gastric ECM or synthetic matrices should cover about 1 to 10 kPa, spanning reported normal and malignant gastric values of roughly 0.5 to 7 kPa (15), and reproduce collagen or hyaluronan composition, oxygen, and flow. Gastric cancer organoid-CAF co-cultures already show stromal effects on chemotherapy response (54). Mechanisms involving T cells, myeloid cells, or checkpoint blockade also require immunocompetent syngeneic models or humanized mice.

Prospective trials should predefine tissue and liquid pharmacodynamic endpoints. Tissue measures may include spatial niche scores, intratumoral drug distribution, collagen orientation, perfusion, stiffness, and penetration by CD8 T cells or natural killer cells. Candidate blood markers include collagen-formation or turnover fragments such as PRO-C3 and PRO-C11. A 74-patient gastric cancer conference abstract associated a greater than 20% post-cycle-1 decline in PRO-C3 and PRO-C11 with longer overall survival (55).

Extracellular-vesicle panels could include CAF-associated HSF1-dependent signatures and miR-21. Source deconvolution is essential because macrophage-derived exosomal miR-21 can confer cisplatin resistance in gastric cancer models (28, 56).

Cell-free DNA methylation panels detect gastric cancer-associated differentially methylated regions (57) and may eventually include mesenchymal or fibroblast-associated markers, but no CAF-specific gastric cancer methylation assay has been clinically validated. Liquid markers should complement spatial tissue analysis rather than replace it. Serial endoscopic biopsies add sedation, bleeding, and rare perforation risks, particularly when tumors are friable or patients receive antithrombotic therapy (58, 59). Practical options include window-of-opportunity sampling at diagnostic endoscopy and planned resection, biopsy during clinically indicated restaging endoscopy, and multi-region ex vivo cores collected immediately from surgical specimens. Biopsy sites should be registered to endoscopic and pathology maps, with prespecified stopping rules for sampling-related adverse events.

Adaptive trials should add short stromal therapy only when biopsy, imaging, or liquid markers demonstrate the matched mechanism, limiting exposure and testing biomarker-defined dependence.

## 7 Conclusions

GC fibroblasts are dynamic components of the treatment response, but they do not uniformly promote resistance. Enrichment after therapy may reflect transition, reversible reprogramming, recruitment, or selection. Acute interferon, NF-κB, and senescence-associated secretory programs should be distinguished from persistent matrix, vascular, immune, and metabolic states. Mechanical forces, the site of fibroblast origin, and dissociation bias all limit interpretation. Clinical translation will require paired spatial sampling, mechanically appropriate 3D organoid-CAF-immune models, immunocompetent validation, and prespecified pharmacodynamic endpoints. Short, mechanism-matched interventions may reprogram fibroblasts, normalize matrix, block paracrine communication, or localize FAP-directed delivery while preserving fibroblasts that restrain tissue injury or support immunity.
